# Local Circulation Maintains the Coexistence of Lake-dune Pattern in the Badain Jaran Desert

**DOI:** 10.1038/srep40238

**Published:** 2017-01-05

**Authors:** Kecun Zhang, Diwen Cai, Yinhuan Ao, Zhishan An, Zichen Guo

**Affiliations:** 1 Northwest Institute of Eco-Environment and Resources, Chinese Academy of Sciences, Lanzhou 730000, China; 2 University of Chinese Academy of Sciences, Beijing 100049, China

## Abstract

Previous studies proposed various hypotheses to the formation of the mega-dunes and water recharge of the lakes in the Badain Jaran Desert but left the coexistence of lake-dune pattern unsolved. This research found that the local circulation, generated from the differences of thermodynamic properties and the unique landscape settings between lakes and mega-dunes, can be applied to interpret the pattern.

The Badain Jaran Desert, located in western Inner Mongolia of China, is China’s second largest shifting desert and covers an area of 4.92 × 10^4^ km^2^, characterized by coexistence of more than 144 desert lakes and thousands of mega-dunes (Fig. 1) whose heights are generally 200–300 m even over 500 m in the southeastern part of the desert^1^,^2^. The climate herein is extreme continental type controlled by Mongolian High, with cold winter^3^. The annual precipitation is 40–120 mm, mainly falling from June through August and annual potential evaporation is over 2,500 mm. And the mean annual air temperature ranges from 9.8 to 10.2 °C. The mean annual wind speed ranges from 2.8 to 4.6 m s^−1^, with dominant wind direction of northwest and west^3^.

Recently, more and more attention had been paid to the formation mechanisms of the unique landscape but hard to reach a consensus^4^. Previous studies have proposed various hypotheses to explain the formation of the mega-dunes and the water recharge of desert lakes^1^,^2^,^4^,^5^,^6^,^7^,^8^. In addition to these conundrums, that the lakes and mega-dunes coexist for ages in such windy environment is remain unsolved^9^. According to ^14^C-dating of rhizoconcretions (fossilized plant roots coated in calcium carbonate) at the bottom of mega-dunes^10^ and organic sediments in the lake^11^, the ages of mega-dunes and lakes (*e.g.* Lake Sumujilin) are 31,750 ± 485 and 8,390 ± 30 years BP, respectively, which indicates a long lastingly harmonious coexistence of lake-dune pattern. Furthermore, the pattern also appears in the majority desert over the world, *e.g.*, Lake Frome in the Strzelecki Desert^12^, Lake Qarun in the Sahara Desert^13^, Crescent Moon Spring in the Kumtag Desert^14^ and 422 lakes in the Tengger Desert^7^. There is no doubt that sufficient water sources are fundamental to maintain desert lakes, otherwise, it will gradually disappear and be buried by aeolian sand. It confuses us why those desert lakes could coexist harmoniously with around dunes rather than buried by aeolian sands for thousands of years. We tend to interpret the pattern from the perspective of local circulation that generates from the differences of thermodynamic properties and landscape settings between the lakes and mega-dunes.

## Experimental design and research methods

**Figure 1:**
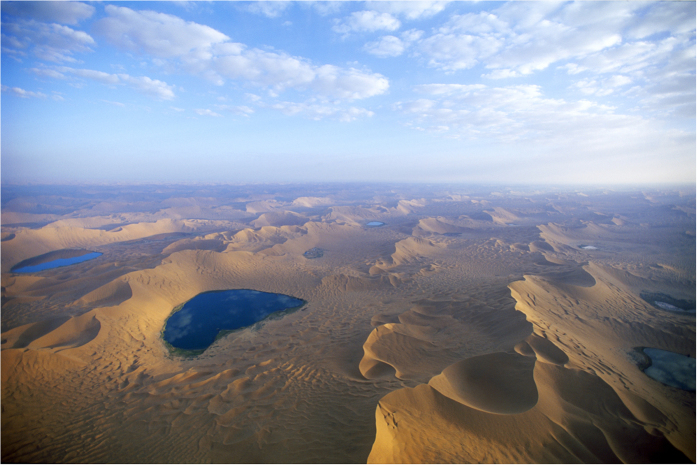
The typical landscape of lake-dune pattern in the Badain Jaran Desert (cited from Dong *et al*.^5^, authorized by Elsevier).

**Figure 2:**
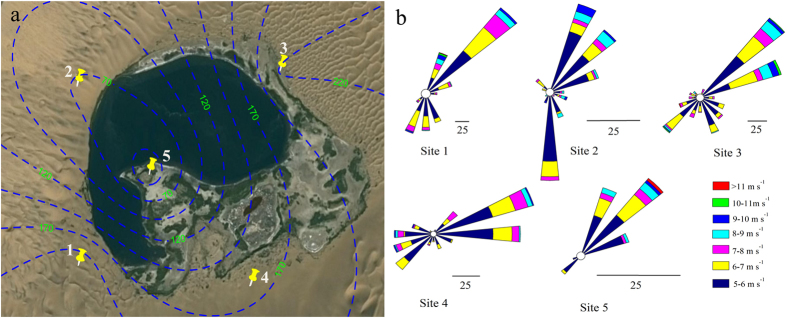
The frequency of sand-laden wind. (**a**) The spatial distribution of the frequency of sand-laden wind (unit: times) around the Lake Yihejidr, the numbers in white and green are observation sites and the frequency of sand-laden wind during the observation period, respectively. And (**a**) was generated by Surfer 8.0 (v11.4.958, http://www.goldensoftware.com/products/surfer). The background image was created by screen-shot from Google Earth (v7.1.5.1557, https://www.google.com/earth/), and the source image was provided by ©2016 CNES/Spot Image DigitalGlobe. (**b**) The frequency roses of segmented sand-laden wind, site numbers are consistent with (**a**), and note the different scales. (**b**) was created by OriginPro 9.0 (v9.0.0.87, http://www.originlab.com/). The merging of the two figures was by Adobe Photoshop CS6 (v10.0.1, http://www.adobe.com).

The research data were obtained from field observation at the Lake Yihejidr (102°8.9′E, 39°46.1′ N; 1,158 m asl), a typical lake in the Badain Jaran Desert. The lake area is about 1.4 km^2^, with maximum dimension of 1.6 km in length and 1.1 km in width (measured from Google Earth map). Besides, the lake is surrounded by mega-dunes to its northwest and southeast. Compared with the lake surface, the relative elevations of the two mega-dunes are 265.2 m and 336.8 m, respectively. Importantly, the lake is at the foot of the leeward slope (slipface) of the northwest mega-dune. The horizontal distance is about 3736 m between the two mega-dunes. There are abundant superimposed dunes developing on the windward slope (facing to northwest) of the mega-dunes. The top 40 to 60 m of the mega-dunes is the crest zone (the reversing zone, Fig. 1), which is the most active part of the dune, and migrates back and forth in response to the variations in wind direction then forms a reversed slipface^5^. In order to clarify the mechanism of the lake-dune pattern, wind direction and wind velocity are fundamentally required for the view of aeolian geomorphologic research. Therefore, a 3D ultrasonic anemometer (CAST 3, Campbell Corp., USA) was implemented in the center of the lake at site 5, in addition, four automatic meteorological stations (HOBO, Onset Corp., USA) were set in the around dunes as well, site 1 to site 4 (Fig. 2a). All instruments were set at a height of 2.0 m above the ground and the intervals of data logging were 15 min. The field observation was carried out from July 16 to September 15, 2009, in which the background wind velocity was relatively low^9^.

## Research results

### Characteristics of sand-laden wind

The sand-laden wind, exceeding threshold velocity to move sand grains (here known as 5.0 m s^−1^), is the basic force to the formation of aeolian geomorphology^15^. During the observation period, the frequency of sand-laden wind decreased rapidly from the center of the lake to the peripheral dunes (Fig. 2a). Additionally, the frequencies of segmented sand-laden wind in the center of the lake are also less than those in the around dune sites (Fig. 2b). The above results indicate that the wind energy is relatively low in the lake area. Actually, owing to the unique settings of the lake and dune, the lake is sheltered by the around dunes from wind erosion.

### Divergence

The local circulation is very common over heterogeneous surfaces, such as urban heat island circulation, land-sea breeze and mountain-valley wind. The mechanisms for local circulation are the differences of thermodynamics and kinetics over heterogeneous surfaces^16^. It is natural to associate the lake-dune pattern with land-sea or mountain-valley pattern. Therefore, we wonder whether there is a specific relationship between the lake-dune pattern and the local circulation. Field observation found that the wind directions were opposite in the center of the lake during the day and night. The horizontal air divergence (*D*) can be used to identify the flow field condition^17^, written by,


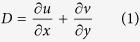


where *D* is divergence, s^−1^. When *D* < 0, it is convergent; when *D* > 0, it is divergent. *u* and *v* is wind velocity in horizontal (*x*) and vertical (*y*) direction, respectively, m s^−1^.

**Figure 3:**
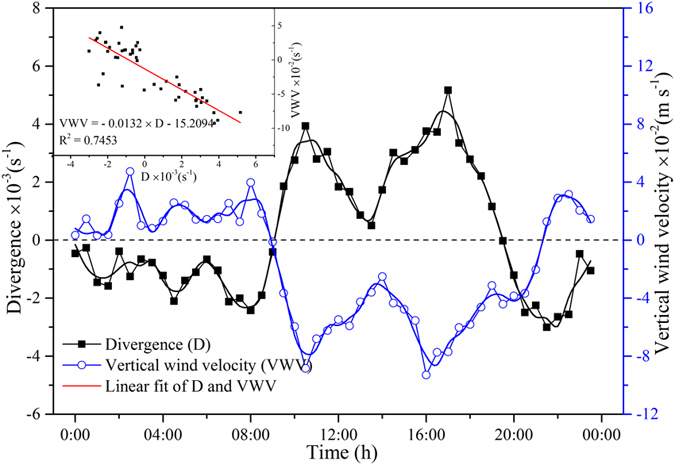
The divergence and vertical wind velocity over the Lake Yihejidr; data collected from the site 5. This figure was created by OriginPro 9.0 (v9.0.0.87, http://www.originlab.com/).

In the center of the lake, the divergence is positive in the daytime but negative at night while vertical wind velocity is just the opposite (Fig. 3), which indicates that the airflow is divergent in the daytime but convergent at night. Moreover, the inlet figure in Fig. 3 presents the relationship that vertical wind velocity negatively decreases with divergence (R^2^ = 0.7453, p_2_tailed_ = 0.01, n = 48, tested by IBM SPSS statistics 22.0, v22.0.0.0.202, http://www.ibm.com/analytics/us/en/technology/spss/). In other words, the wind blows from the lake to the mega-dune in the daytime but opposite at night, which is typical mountain-valley circulation. And Fig. 3 also shows that the vertical wind velocity in the daytime is higher than that at night. Importantly, this means the “valley wind” velocity is higher than “mountain wind”. The above analyses imply the higher capacity of sand transport of “valley wind”, which process may be the reason why the lakes have not been buried by sand.

## Discussion

**Figure 4:**
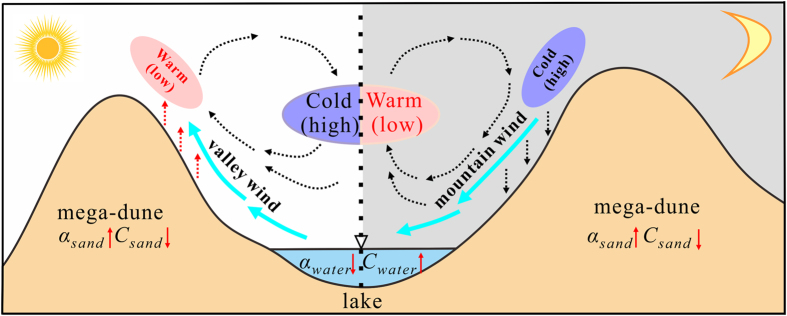
The local circulation during the day (left) and at night (right) between the lakes and mega-dunes. The purple and pale red oval represent relatively cold air (high air pressure) and warm air (low air pressure), respectively. And the dash arrow represents the motion direction of the air. The fluorescent green solid arrow represents the direction of the wind. And the red solid arrow and its direction denote relative value between sand and water. This figure was generated by CorelDRAW X6 (v3.0.1.684, http://www.corel.com/cn/).

In fact, three keys bear the evidences to the coexistence of lakes and mega-dunes in desert environment (Fig. 4). Firstly, the relative relief itself between mega-dunes and inter-dunes in which lake basin lies will generate mountain-valley wind in some circumstances. Generally, the air is mainly heated by long wave radiation emitted from the ground. On a sunny day, the air above the slope of mountain could obtain more heat from the sand surface than the air above the valley at the same height. Consequently, the air above the mountain rises faster than that above the valley, and low air pressure occurs in the near surface of mountain while relatively high air pressure appears in the near surface of valley. Where there is the air pressure difference, there will be air movement, and valley breeze generates. But the case is opposite at night (similar to Fig. 4). This inspires us that the lake-dune pattern is very similar to the mountain-valley pattern. Given the prerequisite that the lake-dune pattern has similar thermodynamic properties with mountain-valley pattern, there must be the same process appearing between them. Besides, our field observation is a good example to testify it.

Secondly, the huge differences of reflectivity (*α*) and specific heat (*C*) between water and sand will strengthen the local circulation. Although the reflectivity of sand is higher than lake water (*α*_*water*_ = 0.25, *α*_*sand*_ = 0.3), the specific heat of lake water is about 4.35 times of sand (*C*_*water*_ = 4.2 × 10^3^ J kg^−1^ °C^−1^, *C*_*sand*_ = 0.97 × 10^3^ J kg^−1^ °C^−1^). In order to clarify whether the lake-dune pattern strengthens the mountain-valley breeze, here comes a semi-quantitative analysis. Importantly, two assumptions are needed to simplify the actual underlying surface conditions: (1) take the lakes and mega-dunes as homogeneous surface, namely water and sand, respectively; and (2) as for comparing with normal mountain and valley, their underlying surfaces are very complicated, therefore, we assume that only includes vegetation, rock and bare soil. The specific heat of normal mountain (*C*_*nM*_) and normal valley (*C*_*nV*_) will be calculated as follows,


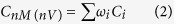


where *ω*_*i*_ and *C*_*i*_ denote weight percentage and specific heat of *i*, and *i* represents the units comprised in underlying surface of normal mountain or normal valley. Moreover, the vegetation, rock and soil are also mixed with various substances, which still follows the equation (2). Take soil as example, it consists of mineral materials, water, humus and microorganisms, thus its specific heat varies greatly with different types of soil. That is why scholars would like to measure it according to actual situation. But as is known to all that the specific heat of water is generally the largest among ordinary substances. Therefore, it is clear that the specific heat of soil is less than that of water through equation (2), and so do the vegetation and rock. Consequently, *C*_*nV*_ is less than *C*_*water*_, which signifies that the lake surface is colder than natural valley with higher air pressure. While there are some differences with *C*_*nM*_. Sand could be reckoned as one of the components of soil, and generally, the surface sand has very low water content during sunny day. This indicates that the sand has lower specific heat addition of water that owns the largest specific heat. Therefore, it is clear that *C*_*sand*_ is less than *C*_*nM*_, which implies that the near surface of sand dunes is hotter than natural mountain with lower air pressure. And which was testified by our field observation. Given identical condition, the average air pressure in the lake area is 2.43 kPa higher than that in the dune area during the observation period. Generally^18^, the mean speed of valley wind is 3~4 m s^−1^, and even up to 6~8 m s^−1^, while 9~10 m s^−1^ is of high frequencies during observation (Fig. 2b). In summary, the lake-dune pattern actually strengthens the local circulation (mountain-valley wind).

Thirdly, the local circulation between lakes and mega-dunes varies greatly with weather and season. The local circulation is of obvious effect on a sunny day and also in summer while relatively weak on a cloudy day and in winter, because the local circulation strongly depends on the thermodynamic differences between lakes and mega-dunes which relies on the energy indirectly from the sun. Besides, it also varies during a single day. The divergence is of relatively high value during daylight with relatively high vertical wind speed while it is just the opposite at night (Fig. 3), which indicates, probably the most valuable point, the mountain wind is lower than valley wind which balances the effect of sand transport by background wind. The diurnal and seasonal wind direction alternation will balance the back and forth sand transport processes. In addition, the crest zone (reversing zone), the top 40 to 60 m of the mega-dunes, is the best evidence to prove it.

Consequently, the effects of local circulation provide an optional interpretation of the coexistence of lake-dune pattern in the Badain Jaran Desert. Though more evidences need to be identified to fully reveal the lake-dune pattern, this finding contributes one small step to the final answer. Local circulation generates from the relatively relief and thermodynamic properties between lakes and mega-dunes. This inspires us more attention should be paid to local circulation in the research of aeolian geomorphology because it is of great importance in some circumstances like lake-dune pattern and pyramidal dunes. Further improvements are expected with combined numerical simulation and by taking into account information on the relationship between local circulation and the large-scale flow pattern.

## Additional Information

**How to cite this article**: Zhang, K. *et al*. Local Circulation Maintains the Coexistence of Lake-dune Pattern in the Badain Jaran Desert. *Sci. Rep.* **7**, 40238; doi: 10.1038/srep40238 (2017).

**Publisher's note:** Springer Nature remains neutral with regard to jurisdictional claims in published maps and institutional affiliations.
